# Patient Experience and Perspective on Medical Cannabis as an Alternative for Musculoskeletal Pain Management

**DOI:** 10.5435/JAAOSGlobal-D-22-00055

**Published:** 2022-07-06

**Authors:** Danny Mangual-Pérez, Ruben Tresgallo-Parés, Manuel Ramírez-González, Norberto J. Torres-Lugo, Asdrúbal Rivera-Dones, Gustavo Rivera-Rodríguez, Alexandra Claudio-Marcano, Luis Lojo-Sojo

**Affiliations:** From the Department of Orthopaedic Surgery, University of Puerto Rico, Medical Sciences Campus, San Juan, Puerto Rico (Dr. Mangual-Pérez, Dr. Tresgallo-Parés, Dr. Ramírez-González, Dr. Torres-Lugo, and Dr. Lojo-Sojo); the School of Medicine, Universidad Central del Caribe, Bayamon, Puerto Rico (Dr. Rivera-Dones and Dr. Rivera-Rodríguez); and the School of Medicine, University of Puerto Rico, Medical Sciences Campus, San Juan, Puerto Rico (Dr. Claudio-Marcano).

## Abstract

**Introduction:**

The current rate of opioid prescription is disquieting because of their high abuse potential, adverse effects, and thousands of overdose deaths. This situation imposes urgency in seeking alternatives for adequate pain management. From this perspective, this study aimed to evaluate the experience and the perceived analgesic efficacy of medical cannabis in managing the pain associated with musculoskeletal conditions.

**Methods:**

A 28-question survey was distributed to patients at a major medical cannabis center in Puerto Rico for 2 months. Demographics, medical history, cannabis usage, cannabis use perspective, and analgesic efficacy were assessed in the questionnaire.

**Results:**

One hundred eighty-four patients completed our survey. The majority (67%) were males, and the participants' average age was 38 years. This study showed an average pain reduction score of 4.02 points on the Numeric Rating Scale among all the participants. Those with musculoskeletal conditions reported a notable average pain reduction score of 4.47 points. In addition, 89% of the participants considered medical cannabis to be more effective than narcotics for adequate pain management.

**Conclusions:**

This study demonstrated that the use of medical cannabis among patients with musculoskeletal conditions effectively reduced pain levels based on their Numeric Rating Scale reported scores.

The increasing use of opioids for pain management has raised concerns about their potential abuse and dangerous adverse effects.^1^ Currently, the United States accounts for 80% of the global opioid consumption,^1^ generating a narcotic crisis that costs approximately $78.5 billion annually.^2^ This situation has led to an increment in the number of deaths secondary to opioid overdose, with more than 750,000 deceases in the United States since 1999.^3^ In addition, a 1.68 million person-year life loss was reported in 2016, with the highest incidence among people of the age of 24 and 25 years (20% of the deaths).^4^ Contrarily, opioid misuse is another critical concern, with statistics ranging from 21% to 29% in patients with chronic pain and 4% to 6% in patients transitioning to heroin use.^5^ Persistent opioid use has been associated with reasons other than the intensity of the surgical pain, generating a harmful situation for the patients due to the disproportionate use of these narcotics.^1^

Orthopaedic surgeons represent close to 10 percent of opioid prescriptions.^6^ These drugs are frequently provided after orthopaedic interventions because of their notable postprocedural pain. Especially considering that adequate postoperative pain management is a major determinant of patient satisfaction.^7^ Therefore, potent narcotics such as morphine, Vicodin, and Percocet are frequently offered to achieve adequate pain suppression. Unfortunately, these medications have a notable risk for addiction and have been associated with adverse effects such as nausea, vomiting, constipation, and respiratory depression. In 2020, Nazzal^6^ reported no increase in pain scores or opioid consumption with prescription of lower doses of oxycodone after anterior cruciate ligament reconstruction, suggesting that achieving adequate pain control with lower doses is feasible.

Finding non-narcotic alternatives with a better safety profile has been a research challenge. Previous studies have contemplated viable options to substitute opioids for pain management.^8-10^ In 2020, Sabesan et al^9^ evaluated multimodal pain management as a possible alternative after shoulder arthroplasty. Their protocol consisted of using gabapentin, acetaminophen, and an ultrasonography-guided interscalene block with 0.5% ropivacaine preoperatively, followed by intraoperative Decadron, ketorolac, and local infiltration of liposomal bupivacaine. The study concluded that the complete elimination of opioid use by two weeks could be achieved with nonopioid-based alternative pain management because patient satisfaction did not diminish. In another study, a 50-mg intravenous (IV) tramadol dose was shown to be safe and well tolerated for postoperative pain management, where traditional IV opioids are often used.^8^ Tramadol is classified as a Schedule IV drug, representing a less severe abuse potential when compared with Schedule II drugs such as morphine and Percocet.^8^ Therefore, IV tramadol could be a temporary alternative over more potent narcotics until better options are found.

Among the potential opioids surrogates, cannabis has been a poorly studied alternative because of its social stigma and limited access. Nonetheless, cannabis has gained considerable attention as a treatment alternative for different diseases such as multiple sclerosis, cancer-related emesis, anorexia, and cachexia.^11-13^ The principal compounds that have been associated with its therapeutic potential are cannabinoids: tetrahydrocannabinol (THC) and cannabidiol. These constituents are found in plants belonging to the Cannabis genus, such as *Cannabis indica*, *Cannabis sativa*, and *Cannabis ruderalis*. Cannabinoids are classified as phytocannabinoids derived from plants, endocannabinoids are produced endogenously in the body, and synthetic cannabinoids are created artificially.^14^

The endocannabinoid system has shown promising results as an analgesic and anti-inflammatory pathway.^15^ Other functions associated with this complex system are metabolic regulation, appetite, blood pressure, and the sense of reward.^16,17^ THC, the primary psychoactive component of cannabis, has demonstrated effectiveness in pain suppression, appetite stimulation, and digestion, acting on the cannabinoid receptors CB1 and CB2. CB1 receptors are associated with nociceptive areas of the central and peripheral nervous systems while CB2 receptors have shown the capability to suppress pain and inflammation.^18^ Therefore, some studies have focused on patients with inflammatory arthropathies and musculoskeletal pain.^15^

Medical cannabis can be administered through different methods; the most common routes are inhalation and oral ingestion. Moreover, cannabis could be administered through lotions, cartridges, concentrates, and sublingual drops.^19^ Medical cannabis has been recently known for its antiemetic, appetite stimulant, and analgesic therapeutic properties. In 2017, Romero-Sandoval et al^20^ evaluated the analgesic effects of medical cannabis in patients with chronic pain. They concluded that inhaled cannabis with a THC concentration of 7% (28 mg) was the most effective in reducing chronic neuropathic pain. However, additional studies are necessary to understand the use and efficacy of medical cannabis as an adequate therapeutic alternative in pain management.

Many strategies have been studied to reduce postoperative pain and avoid the opioid crisis, yet cannabis remains poorly assessed.^15^ In this perspective, we developed a questionnaire to evaluate the experience and the perceived analgesic efficacy of medical cannabis in the management of the pain associated with musculoskeletal conditions. This study aims to elucidate whether medical cannabis represents a satisfactory alternative for pain management in musculoskeletal disorders in a Hispanic population.

## Methods

A cross-sectional study was designed to assess the perceived efficacy of cannabis as an analgesic alternative in musculoskeletal conditions. The study population consisted of patients of a major medical cannabis center in Puerto Rico with multiple locations throughout the island. The dispensaries operate under the 42 to 2017 Law of the Government of Puerto Rico to market cannabis and its derivates for medical purposes, the sole approved use in the territory. All patients of the dispensaries meet the inclusion criteria consisting of age 21 years and older and having a valid license for medical cannabis use. However, patients of other medical cannabis centers were excluded to ensure that their products do not have a different formulation than the selected dispensaries. Enrollment in this study was voluntary and subject to written informed consent before participating.

**Figure 1:**
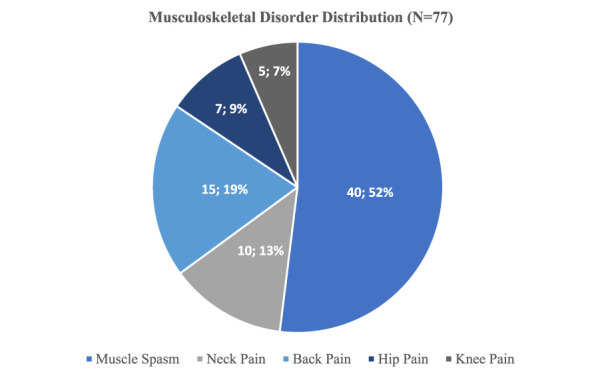
Pie chart demonstrating the distribution of conditions on the musculoskeletal disorders subset.

We prepared a 28-question survey that was reviewed and approved by the senior author **(**Figure 1**)**. The anonymous questionnaire was administered at the dispensaries for a participation period of two months. The research personnel approached potential participants at the dispensaries' security checkpoint (verify for valid cannabis license) and extended an invitation to participate after fully disclosing the study objectives, including risks and benefits. Survey sheets were provided to all the patients who gave consent with instructions to fill the document and return it at the end of their appointment.

The data assessed in the questionnaire included demographics, medical history (medical conditions, allergies, and medications), toxic habits (tobacco, alcohol, or non-cannabis drugs), cannabis usage questions, cannabis perspective questions, and an analgesic efficacy evaluation using the Numeric Rating Scale (NRS). The NRS score before cannabis use and the NRS score reported after cannabis use were compared to assess the analgesic efficacy of cannabis as a potential alternative for pain management. In addition, information regarding their last four digits of phone number and initials were requested to avoid multiple participation because some patients frequently visit these dispensaries.

Statistical analysis was conducted using IBM SPSS software. Categorical data were evaluated using Pearson chi square and continuous data with analysis of variance. The Fisher exact test evaluated categorical data sets in groups with low-frequency counts. Descriptive analysis was done by analyzing frequencies, mean, and SD. A *P*-value of <0.05 was considered statistically significant. This study was approved by the Institutional Review Board at our academic center.

## Results

### Demographics

Two hundred and thirteen patients meeting our inclusion criteria were invited to participate in this study. A total of 184 patients fully answered our questionnaire (response rate = 86%). The average age of the participants was 38.0 ± 13.0 years (range: 21, 71). They were distributed as 124 males (67%) and 60 females (33%). Their average body mass index was 27.0 ± 6.5 kg/m^2^ (range: 15, 51). The most frequently reported education level was bachelor's degree (79, 43%), followed by high school (65, 35%). Most of the patients (144, 79%) reported a mean annual income of less than $29,999. The demographic data is summarized in Table 1.

**Table 1:** Respondents' Demographics

N = 184	Mean ± SD (Range)
Age	38 ± 13.0 (21, 71)
Sex	Percentage of respondents
Male	67
Female	33
Body measurements	Mean ± SD (range)
Height (in)	67 ± 4.0 (53, 77)
Weight (lbs)	173.1 ± 43.7 (90, 350)
BMI (kg/m^2^)	27 ± 6.5 (15, 51)
Education level	Percentage of respondents
Less than high school	3
High school	35
Associate degree	12
Bachelor's degree	43
Masters degree	5
Doctorate degree	2
Annual income	Percentage of respondents
Less than $9999	38
$10,000-$29,999	41
$30,000-$49,999	13
$50,000-$69,999	5
$70,000-$89,999	2
$90,000 or more	2

### Medical History and Toxic Habits

Seventy-seven of the participants reported chronic medical conditions, with the most reported being hypertension (35; 19%), diabetes (17; 9%), hypothyroidism (14, 8%), and fibromyalgia (11, 6%). Eighty-six patients (47%) reported alcohol consumption among the study participants while 48 patients (26%) reported tobacco use. Among the noncannabis drug use, the prescribed narcotics for pain include tramadol (47%), Percocet (43%), codeine (23%), and Ultracet (7%) (Table 2). Moreover, 31% of these participants reported using any previously mentioned narcotics with medical cannabis.

**Table 2:** Medical History and Toxic Habits

Chronic Medical Conditions	Percentage of Respondents
Hypertension	19
Diabetes	9
Hypothyroidism	8
Fibromyalgia	6
Toxic habits	Percentage of respondents
Alcohol	47
Tobacco use	26
Previously prescribed narcotics	Percentage of respondents
Tramadol	47
Percocet	43
Codeine	23
Ultracet	7

### Indications for Medical Cannabis Use

The reported medical indications for cannabis use were extensive. The reported conditions include anxiety disorders (115), insomnia (100), and musculoskeletal disorders (77) (Table 3). These values are consistent with the fact that more than one condition could be included when licensing for medical cannabis. Among the participants, 41.8% of the patients reported musculoskeletal disorders. Those patients with orthopaedic conditions (ie, musculoskeletal disorders) include muscle spasms (40, 52%), lower back pain (15, 19%), neck pain (10, 13%), hip pain (7, 9%), or knee pain (5, 7%). They were considered a subgroup for additional analysis (Figure 1). The most common indication for medical cannabis use within this group was muscle spasms (40, 52%).

**Table 3:** Respondents' Medical Indications for Cannabis Use

Conditions	Reported Frequency	Percentage of Respondents
Anxiety	115	63
Insomnia	100	54
Musculoskeletal disorders	77	42

### Medical Cannabis Products

**Figure 2:**
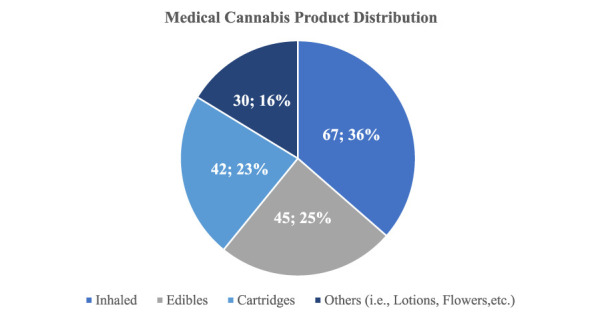
Pie chart demonstrating the distribution of medical cannabis product formulations used by the respondents.

The most used cannabis products were inhaled formulations (67, 36%), followed by edibles (food products infused with cannabis; 45, 25%) and cartridges (42, 23%; Figure 2). However, in the musculoskeletal disorder subset, the use of cannabis-based lotions was significantly higher (*P* < 0.028) when compared with the rest of the patients. The reported monthly expenses on medical cannabis products average 331 ± 278 dollars (range: 278, 2,040) with no notable difference when evaluated in the musculoskeletal condition subset.

### Perspective on Medical Cannabis Use

Among the participants, 164 patients (89%) considered cannabis more effective than narcotics (ie, opioids) for pain management. Moreover, 165 participants (90%) will recommend cannabis over prescribed drugs. All the patients (100%) reported feeling safe buying medical cannabis in a dispensary. One hundred sixty-six participants (90%) considered that medical cannabis should be used in a hospital scenario (inpatient). Furthermore, the majority (91%) reported that medical cannabis should be covered by health insurance (Table 4).

**Table 4:** Answers to Questions on the Perspective on Medical Cannabis Use

Questions	Yes	No
Do you think medical insurance should cover the cost of medical cannabis products in the future?	91%	9%
Do you think hospitals should allow or consider the use of cannabis products while being hospitalized?	90%	10%
Do you feel safe buying medical cannabis products at a dispensary?	100%	0%
Do you think medical cannabis control pain more effectively than narcotics?	89%	11%
Do you recommend the use of medical cannabis over other analgesics and medications?	90%	10%

### Medical Cannabis Analgesic Efficacy

NRS was used to assess and compare pain levels before and after using medical cannabis. The average pain reduction of the study participants was 4.02 points based on the NRS reported score. The evaluation of the musculoskeletal disorder subset showed a significantly higher average pain reduction score (4.47; *P* < 0.001). Contrarily, when the musculoskeletal disorder subset was removed from the data set, the average pain reduction score was lower but remained statistically significant (3.70; *P* < 0.001; Table 5). Daily cannabis use was reported in most patients (90%), with no significant difference when analyzed within the study subsets.

**Table 5:** Average Pain Scores on the Numeric Rating Scale (NRS)

All Participants (N = 184)				
NSR Score	Before Medical Cannabis	After Medical Cannabis	Pain Reduction Score	*P* Value
Average scores	7.43	3.41	4.02	<0.001
Participants without musculoskeletal disorders (N = 107)				
NRS score	Before medical cannabis	After medical cannabis	Pain reduction score	*P*-value
Average scores	6.96	3.26	3.7	<0.001
Participants with musculoskeletal disorders (N = 77)				
NRS score	Before medical cannabis	After medical cannabis	Pain reduction score	*P*-value
Average scores	8.09	3.62	4.47	<0.001

## Discussion

Our results demonstrate that medical cannabis patients perceived this drug (ie, cannabis) as an effective pain management alternative for musculoskeletal pain based on their NRS reported scores. In addition, most participants perceived medical cannabis as a better alternative than narcotics to attain adequate pain control.

### Opioids Pandemic: Could Medical Cannabis Be a Potential Alternative?

Currently, the dependence and abuse of opioids are a growing dilemma. It has been estimated that 150 million opioids are prescribed annually in the United States, an increasing problem over the past 20 years.^3^ Owing to their potent analgesic properties, opioids have been the drug of choice by orthopaedic surgeons to manage postoperative pain.^7^ Despite their known risks, avoiding opioid use has been a challenge because of the high pain rates in the postoperative period. Therefore, exploring effective and safer alternatives is crucial to fighting the opioid pandemic.

Medical cannabis is a promising alternative for replacing opioids. Owing to its theoretical anti-inflammatory and analgesic properties, this group of compounds (ie, cannabinoids) represents a potential pain management strategy.^20,21^

### Current Applications of Medical Cannabis

Cannabis is a drug that has become increasingly popular in recent years. Despite their Schedule 1 drug category, it is important to note that certain states have legalized both recreational and medicinal use of cannabis. Knowing the most common reasons for cannabis use can provide valuable information about the most common tendencies for using this drug and further determine its effectiveness. In a study done by Cahill et al,^22^ the most common diseases for which the participants reported cannabis use were anxiety (15.4%), followed by posttraumatic stress disorder (15%), rheumatoid conditions (13.1%), sleep disorders (12.6%), and degenerative disk disorders (10.3%) among others. In a cross-sectional study by Lucas et al,^19^ chronic pain (29.4%) was the most common indication for medical cannabis use, followed by mental health conditions (27%), insomnia (9.7%), musculoskeletal conditions (9.4%), and posttraumatic stress disorder (4.6%) among others. Those results have certain similarities to our findings, where the most common reasons for medical cannabis use were anxiety, insomnia, chronic pain, and muscle spasms. Moreover, chronic pain (88%) (ie, knee pain, back pain, and neck pain) was the most common reason in the musculoskeletal disorders subgroup.

### Cannabis Usage and Implications

In the perioperative setting, the surgeons and anesthesiologists must consider all aspects of cannabinoids usage. Anecdotal reports have detailed high anesthetic requirements, and a recent study reported an increase in propofol doses required to achieve successful laryngeal mask insertion and intubation.^23^ Some reports have described the incidence of coronary ischemia, myocardial infarction, pulmonary edema, and cerebral ischemia even in young adults, although these are rare.^24,25^

Some studies describe the effects of cannabis use in patients undergoing hip and knee arthroplasty. They report increased complication rates characteristic of IV drug administration as opposed to inhalation or ingestion.^26,27,28,29^ Another study analyzing drug misuse describes increased surgery-related complications and extended hospital stays in substance-abusing patients who undergo total hip and knee arthroplasties. However, this study was not limited to cannabis use alone.^26^ Prior research using the National Hospital Discharge Survey investigating drug misusers (opioid, cocaine, cannabis, amphetamines, inhalants, and sedatives) who underwent primary total knee arthroplasty or total hip arthroplasty found that drug misusers had higher rates of surgery-related complications (*P* < 0.001).^26^ The authors reported a higher rate of periprosthetic joint infections (1.6% compared with 0.1%; *P* < 0.001) in the drug misusers group.^26^ The THC immunosuppressive effect has been hypothesized to impair the release of proinflammatory cytokines and therefore weaken the immune response.^30^ Contrarily, this immunosuppressive effect modulates the inflammatory response, which is lowered in arthritic joints.^31^

Law et al^32^ showed a lower rate of periprosthetic fracture, mechanical loosening, implant failure, and osteolysis as a cause of total knee arthroplasty revisions in the cannabis-user group. These findings did not reach statistical significance, which can be partially explained by studies investigating the effect of upregulating cannabinoid receptors (CBs) of the skeleton in mice. Bab et al^33^ showed that THC activates CB2, a CB expressed on osteoblasts and osteoclasts. Activating the CB2 receptor stimulated bone formation, balanced bone remodeling, and perhaps played a protective role against age-related bone loss.^34^ A clinical cross-sectional study of 109 heavy cannabis users (mostly young men) compared with non-heavy cannabis users showed substantially lower bone mineral density Z-score values at the lumbar spine and hip evident on dual-energy X-ray absorptiometry (DEXA) scans on the heavy cannabis users.^35^

A recent review analyzed data from the Healthcare Cost and Utilization Project Nationwide Inpatient Sample database to evaluate the effect of marijuana use by orthopaedic patients on inpatient mortality, heart failure, stroke, and cardiac disease. A decreased mortality rate was seen in patients who used marijuana.^36^ This study did not stratify by comorbidities nor by demographics of marijuana users.

Clinical trials have investigated some of the most common indications for the therapeutic use of cannabinoids, including posttraumatic stress disorder, anxiety, sleep, and schizophrenia. Currently, there is insufficient evidence to estimate efficacy in these areas. Among the cannabis indications examined, acute and chronic pain management remains the most pertinent for orthopaedic surgery patients. In the US pain clinics, up to 80% of the cannabis users report myofascial pain as their primary diagnosis.^16^ In this perspective, the use of cannabinoids based on their specific analgesic properties may represent the most promising indication for their clinical integration. This application represents a vital asset in the fight against the opioid crisis.

### Perceived Cannabis Efficacy as an Alternative for Pain Management

Opioid use for pain management is a critical component in postoperative settings. However, there is an urgent need to seek less dangerous and addictive drugs. Corroborating the efficacy of medical cannabis compared with opioids is necessary to establish a proper relationship between cannabis and its analgesic effects. A study conducted by Perron et al^37^ showed that efficacy ratings for pain management among cannabis users were higher compared with prescription pain medication users. Using an NRS between 0 and 10 (0 = not helpful, 10 = very helpful), Perron et al^37^ determined that cannabis was more efficacious with a mean score of 7.57 versus those on prescription pain medication who had a mean score of 5.31.

By contrast, a pilot study conducted by Romero-Sandoval et al^20^ demonstrated that oral THC had no effect on opioid consumption and had no positive effect on pain levels. After surgery, participants reported that consuming a synthetic cannabinoid drug (nabilone) produced higher pain scores than placebo. According to Lucas et al,^19^ 610 participants in their study who substituted cannabis for opioids reported a 59.3% complete cessation of opioid use, whereas 18.4% of the participants reduced opioid use by 75%. The main reasons for using cannabis over opioids were that patients considered cannabis to be a safer alternative to prescription drugs (51.2%), followed by better symptom control (19.5%). Similar to the studies by Perron et al^37^ and Lucas et al,^19^ our study demonstrated an overall pain reduction of 4.02 points, with a musculoskeletal disorder subset showing an even higher average pain reduction score of 4.47 points.

In 2020, Brady et al evaluated the self-efficacy of medical cannabis patients in the context of researching, procuring, and using cannabis. Their study defines self-efficacy as an individual's belief in their capacity to produce a certain outcome. In this context, he observed that those patients with higher self-efficacy about medical cannabis were more likely to engage in activities to experiment and incorporate medical cannabis into their medical management despite the perspective of their providers about this treatment modality.^38^ These findings may provide a take into our results that 89% of the assessed medical cannabis patients considered that cannabis is better than opioids for their pain management.

### Limitations

This study has some limitations. First, using a questionnaire as a research tool imposes intrinsic restrictions such as differences in questions interpretation, dishonesty, unconscientious responses, or uncertain answers. Second, because more than one condition could be reported for medical cannabis licensing in our territory, there were some constraints in evaluating a group limited solely to musculoskeletal disorders. Third, the reported VAS score could have been affected by recall bias. Finally, medical cannabis is produced in varying quality, consumed in different quantities, and available through different brands providing some heterogenicity between patients that could influence the perceived efficacy among the participants. Future studies should assess these heterogenic features (ie, dosages, quality, and brands) to guide medical professionals toward a more accurate prescription for medical cannabis.

## Conclusion

Medical cannabis is an understudied drug with theoretical analgesic properties that could represent an asset against the current opioid pandemic. This study showed that the use of medical cannabis among patients with musculoskeletal conditions effectively reduced pain levels based on their NRS reported scores. In addition, most patients using medical cannabis considered that this drug represents a better option than narcotics (ie, opioids) for adequate pain management. Additional studies on medical cannabis should evaluate whether the experience and perspective presented through this study could translate into satisfactory and consistent clinical outcomes.
